# Evaluation of Metal Oxide Surface Catalysts for the Electrochemical Activation of Amino Acids

**DOI:** 10.3390/s18093144

**Published:** 2018-09-18

**Authors:** Christian A. Tooley, Charles H. Gasperoni, Sabrina Marnoto, Jeffrey Mark Halpern

**Affiliations:** Department of Chemical Engineering, University of New Hampshire, Durham, NH 03824, USA; christianatooley@gmail.com (C.A.T.); chg1001@wildcats.unh.edu (C.H.G.); smm1007@wildcats.unh.edu (S.M.)

**Keywords:** sensor, cyclic voltammetry, electrocatalysis

## Abstract

Electrochemical detection of amino acids is important due to their correlation with certain diseases; however, most amino acids require a catalyst to electrochemically activate. One common catalyst for electrochemical detection of amino acids are metal oxides. Metal oxide nanoparticles were electrodeposited onto glassy carbon and platinum working electrodes. Cyclic voltammetry (CV) experiments in a flow cell were performed to evaluate the sensors’ ability to detect arginine, alanine, serine, and valine at micromolar and nanomolar concentrations as high as 4 mM. Solutions were prepared in phosphate buffer saline (PBS) and then 100 mM NaOH. Specifically, NiO surfaces were responsive to amino acids but variable, especially when exposed to arginine. Polarization resistance experiments and scanning electron microscopy (SEM) and energy-dispersive X-ray spectroscopy (EDS) data indicated that arginine accelerated the corrosion of the NiO catalyst through the formation of a Schiff base complex.

## 1. Introduction

Changes in baseline amino acid concentrations are correlated with various diseases such as obesity [1] or major depressive disorder [2,3,4]. Specifically, alanine, serine, arginine, and valine, among others, have been shown to be disrupted in major depressive disorder patients compared to healthy controls [2,3,4]. Monitoring these changes can be done by high performance liquid chromatography–mass spectrometry (HPLC-MS) or nuclear magnetic resonance (NMR) spectroscopy [2,3,4,5,6,7]; limitations of these spectroscopic techniques include low throughput with complicated and/or tedious analysis. These disadvantages are contrasted by electrochemistry; electrochemical detection is highly rapid and sensitive, requiring little sample, and can evaluate the reduction or oxidation of an analyte through the current response [8]. 

Many amino acids lack electrochemically active functional groups. Derivatization is commonly used [9,10], but stability issues are introduced with amino acid derivatives [11]. Furthermore, derivatization can be costly because the product needs to be extracted into organic solvent before analysis [12,13]. A robust sensor would not require extensive sample preparation and analysis would ideally be performed under aqueous conditions. Alternatively, a surface catalyst could be used to electrochemically activate amino acids [14]. Transition metal nanoparticle surface modifications have demonstrated electrocatalytic activity with various amino acids. Amino acids exhibited electro-catalysis with NiO [15,16], CuO [17], and Fe_3_O_4_-graphene oxide nanocomposite [18] surface catalysts. 

**Figure 1:**
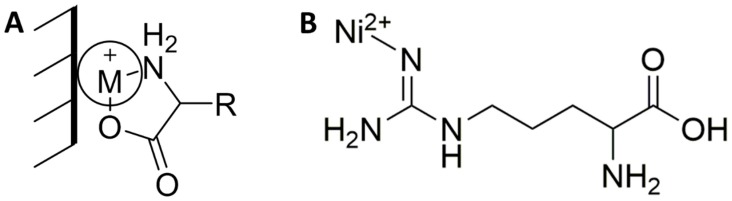
(**A**) Chelation intermediate of amino acid with metal nanoparticle and (**B**) arginine Ni(II) complex through the guanidine moiety.

The mechanism of electrooxidation is not well understood. According to the leading theory, the amino acid chelates to the transition metal nanoparticle to form a catalytic intermediate (Figure 1A) [17]. Chelation only occurs under basic conditions, when the carboxylic acid is negatively charged and the amine has a lone pair to allow complexation (Figure 1A) [19,20]. The chelation intermediate complex allows the metal oxide to catalytically react with the amino acid, presumably through an inner sphere electron transfer, creating an electrochemical measurement. Alternatively, for guanidine derivatives such as arginine, a Schiff base complex can form between the double-bonded nitrogen and metal ion (Figure 1B) [21,22,23]. Since arginine and other structural analogs of alkyl-guanidines are able to complex to various transition metals like Pt(II), the authors propose that a Schiff base complex is formed with the guanidine functional group. If a Schiff base complex is formed, a reverse or variable current vs. concentration would be observed due to the increases of the solubility of the catalyst and the delamination or removal of metal nanoparticles from the electrode surface (Figure 1B).

**Figure 2:**
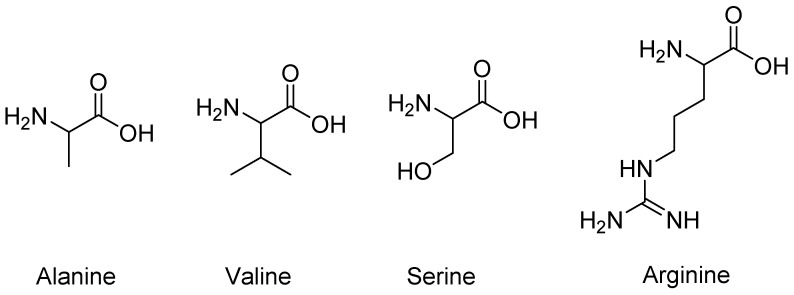
Amino acids that were electrochemically evaluated.

Reported in this study are metal oxide surfaces on glassy carbon and platinum working electrodes and their evaluation as sensors for the detection of alanine, arginine, serine, and valine (Figure 2). Alanine, valine, serine, and arginine are chosen because of their range of pK_a_ values, differences in relative charge and branch groups, and relevancy in various diseases as potential biomarkers [24,25]. Where metal oxides are commonly tested with alanine or serine, they are rarely tested with arginine. The authors used cyclic voltammetry (CV) in a flow cell to electrochemically oxidize the amino acid analytes in 100 mM NaOH solution. They also used corrosion tests and scanning electron microscopy (SEM) and energy-dispersive X-ray spectroscopy (EDS) to evaluate the nickel oxide surfaces to look for any changes before and after amino acid exposure.

## 2. Materials and Methods

### 2.1. Materials and Apparatus

Cyclic voltammetry measurements were recorded on a 600+ Reference Potentiostat (Gamry Instruments Inc., Warminster, PA, USA). Samples were injected into a platinum counterelectrode flow cell with Ag/AgCl reference electrode using a syringe pump (flow rate of 15 μL/h). A dual 3 mm glassy carbon and dual 3 mm platinum working electrode (BASi, West Lafayette, IN, USA) were used in the electrochemistry experiments.

All chemicals were purchased from Fisher Scientific (Bridgewater, NJ, USA), as follows: 2 mM Fe(NO)_3_(*aq*) and 2 mM Ni(NO)_3_(*aq*) solutions were prepared in acetate buffer (pH ~4); 2 mM Cu(SO_4_)_2_(*aq*) solution was prepared in 100 mM Na_2_SO_4_(*aq*); and 4000 μM, 2000 μM, 1000 μM, 400 μM, 200 μM, 100 μM, 75 μM, 50 μM, and 25 μM solutions of alanine, arginine, serine, and valine were prepared by serial dilution in 100 mM sodium hydroxide as well as 10 mM phosphate buffer saline (PBS) solution (pH ~7.4).

A Tescan Lyra3 GMU FIB SEM (Kohoutovice, Czech Republic) was used to obtain the SEM and EDS measurements. A default of a 22 μm view field, at 25.2 k× magnification, and either a 6.0 or 15.0 kV beam was used, indicated on the SEM figures. For EDS measurements, only a 15.0 kV beam was used.

### 2.2. Attachment Method 

The modification procedure followed previously published attachment methods [15,17]. A 2 mM metal ion solution in acidic buffer was syringe pumped through the flow cell at a rate of 15 mL/h. Cyclic voltammetry was performed by sweeping the potential sweep from 0 to −0.8 V at a rate of 100 mV/s for 40 cycles with auto max current. Sodium hydroxide (100 mM) was syringe pumped through the flow cell at a rate of 15 mL/h.

### 2.3. Cyclic Voltammetry and Polarization Resistance Measurements

Cyclic voltammetry was performed by sweeping the potential from 0 to +0.6 V at a rate of 100 mV/s for 40 cycles. Polarization resistance was performed by flowing 100 µM amino acid in 100 mM NaOH at a rate of 1 mL/h over a NiO modified amorphous carbon electrode.

## 3. Results and Discussion

Bare platinum electrodes were tested with the four amino acids which resulted in no electrochemical activity (Figure S1). The working electrodes were modified with a metallic surface by applying a negative potential sweep from 0 to −0.8 V in a metal ion salt solution (Figure S2). A strong cathodic current was measured, which was indicative of electrodeposition of the elemental metal. Passivation to the metal oxide was done in 100 mM NaOH by sweeping the potential from 0 to +0.6 V (Figure S3). For the nickel surface, an oxidation current was measured at approximately +450 mV during passivation and a reduction event occurred at approximately +388 mV, which was indicative of a NiO/NiO(OH) formation.

At physiological pH, catalytic current was not measured presumably because amino acid analytes are unable to chelate at near neutral pH due to protonation of the amine group (Figures S4 and S5). Protonation of the amino group prohibits formation of the dative bond between the nitrogen and metal ion.

### 3.1. Amino Acid Detection at a CuO Surface

**Figure 3:**
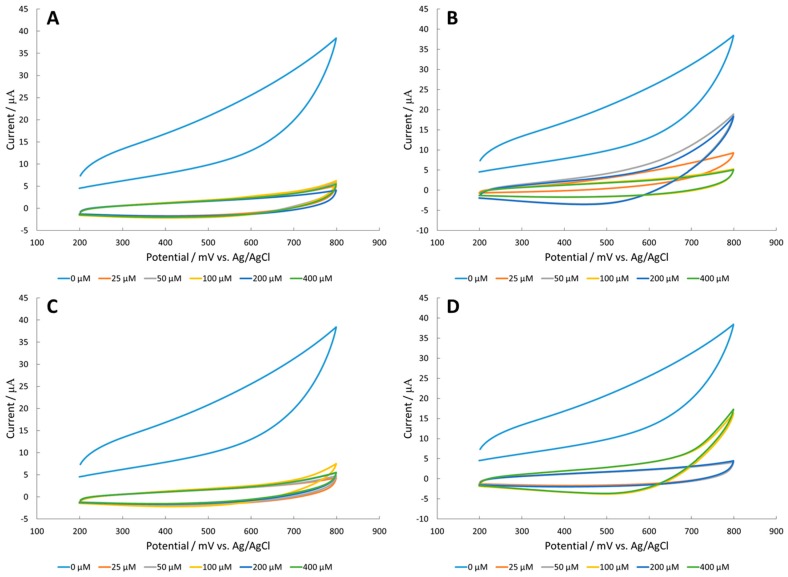
Cyclic voltammetry (CV) of (**A**) alanine, (**B**) arginine, (**C**) serine, and (**D**) valine in 100 mM NaOH with a CuO modified platinum working electrode.

The copper oxide catalyst on the platinum working electrode did not catalyze the electrochemical oxidation of amino acids (Figure 3). Interestingly, the amino acids exhibited lower current measurement than the blank. Luo et al. suggested that amino acid complexation to the electrochemically reduced Cu_2_O surface leads to delamination of the metal oxide surface [26]. In order to obtain a more stable catalyst, the authors attempted to detect amino acids with Fe_2_O_3_ surfaces.

### 3.2. Amino Acid Detection at a Fe_2_O_3_ Surface

**Figure 4:**
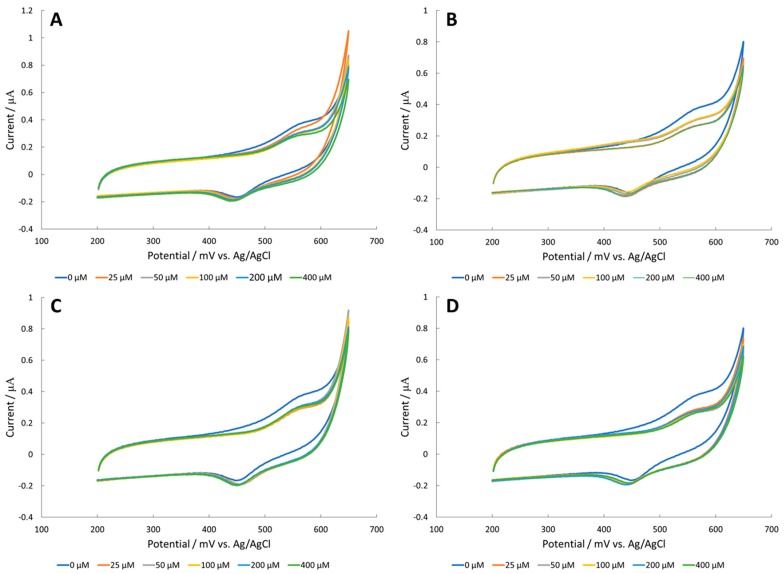
CV of (**A**) alanine, (**B**) arginine, (**C**) serine, and (**D**) valine in 100 mM NaOH with an Fe_2_O_3_ modified glassy carbon electrode.

Reports have demonstrated that amino acids are able to chelate to iron oxide surfaces through vibrational spectroscopy [27,28,29,30]. The Fe_2_O_3_ surface on a glassy carbon electrode exhibited very low current measurements, below 0.6 μA (Figure 4). Furthermore, current measurements decreased in the presence of amino acid analytes. Delamination may occur during amino acid ligation to the iron oxide surface due to increased solubility. According to the Pourbaix diagram, Fe(OH)^−4^ could form on the surface, solubilizing surface iron groups and leading to a decrease in activity over time. The amino acid analytes might be facilitating the dissolution of the iron surface. Therefore, the authors tried NiO surface modifications, which are considered more stable according to the nickel Pourbaix diagram.

### 3.3. Amino Acid Detection at a NiO Surface

**Figure 5:**
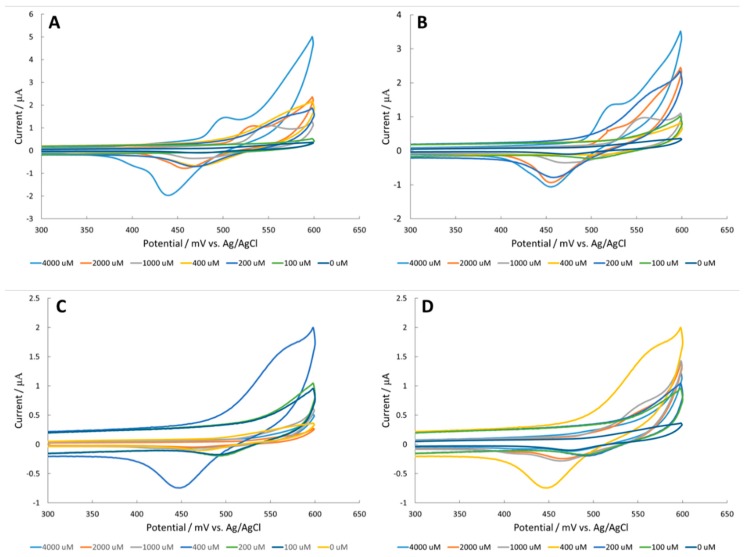
CV of (**A**) alanine, (**B**) arginine, (**C**) serine, and (**D**) valine in 100 mM NaOH with NiO modified glassy carbon electrode.

NiO was reported as a competent catalyst for the electrochemical oxidation of glycine, alanine, serine [15], and arginine [16] between 1 and 400 μM in NaOH. To confirm that the Ni(II) surface did not inactivate or reduce activity with an electrochemical standard, ferrocyanide was measured before and after modification (Figure S6). The Ni(II) surface had the greatest effect on the electrochemical catalytic activity of the amino acids, more specifically arginine (Figure 5). Although activity was observed in the presence of arginine, no clear trend between current and analyte concentration was observed (Figure 5). Alanine and arginine had catalytic activity at 100, 200, and 400 μM, but the signal transitioned to a reversible peak at 1000 μM (1 mM). The detection of serine had concentration-independent electrocatalytic activity at 200 μM and 400 μM, but no activity over 1000 μM. The activity for valine was sporadic, leading to an increase in catalytic activity and reversibility at 400 μM with less activity at higher and lower concentrations. It is possible to get a trend of concentration vs. current signal at one specific potential (amperometric effect). Yet, the overall cyclic voltammetry activity indicates an instable surface, loss of activity (i.e., fouling), or saturation effects. 

### 3.4. Corrosion of Nickel Oxide Surface Measured Through Polarization Resistance and Proposed Mechanism

Polarization resistance experiments were conducted to determine whether the analytes were accelerating the corrosion of the NiO surface. The corrosion rate of the surface was extrapolated from a current resistance vs. potential curve over a stable and controlled region (Figures S6–S9). New surfaces were prepared of the NiO surface to compare the effect of arginine on the nickel surface compared to a buffered solution. Arginine had the greatest effect on voltammograms at (7.8 ± 0.6) × 10^−3^ mpy compared to no analyte present (6.3 ± 0.7) × 10^−3^ mpy (Table 1). The corrosion rate of creatine, which has a guanidine functional group, was also tested and was found at (9.5 ± 1.2) × 10^−3^ mpy.

To ensure that the corrosion was specific to arginine and not inactivity, the authors ran a corrosion rate of arginine and valine in PBS which was found to be 8.5 × 10^−3^ mpy and 4.6 × 10^−3^ mpy, respectively. This indicates that arginine corrodes at a similar rate in both PBS and NaOH. Additional corrosion measurements, measured on already used surfaces, can be found in (Table S1). The data follows the same trend presented above, yet the rates are less accurate because of potential delamination and surface degradation that occurred with prior experiments.

**Figure 6:**
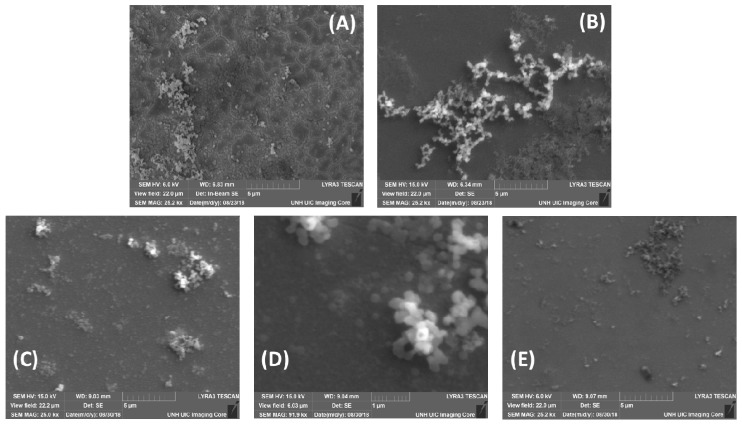
Scanning electron microscopy (SEM) images of (**A**) nickel, (**B**) nickel oxide, (**C**) nickel oxide after phosphate buffer saline (PBS), (**D**), zoomed in on the particle of nickel oxide after PBS, and (**E**) nickel oxide after arginine. There is less nickel oxide content and thickness observed after arginine; this is comparable to original nickel graphs. All images are at 25 k× magnification except (**D**).

SEM images were taken of both nickel (Figure 6A) and nickel oxide (Figure 6B) during the formation process. EDS results indicated the pure nickel composition and NiO composition proposed (see Supplemental Information for the full EDS report). After experiments, surfaces were reevaluated with SEM. The surface exposed to valine in PBS had minimal surface variation (Figure 6C), with the EDS report indicating some PBS salt residue on the surface, but the NiO surface intact. A zoomed-in image of the NiO particles, confirmed with EDS, showed clear round structures (Figure 6D). SEM images after arginine showed less particulate and less nickel oxide content compared to the other surfaces (Figure 6E).

**Figure 7:**
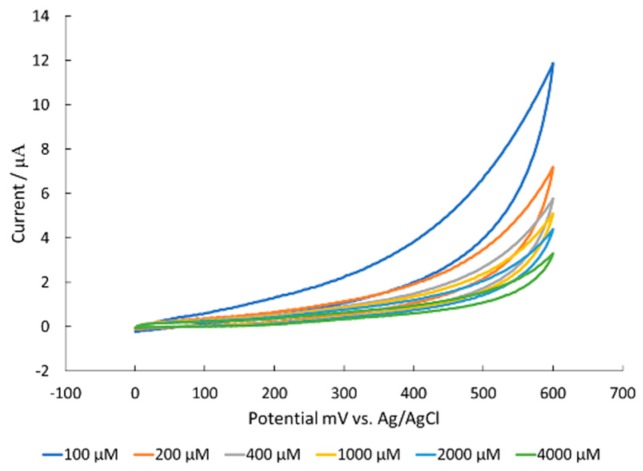
CV data of a serial dilution of creatine in 100 mM NaOH with a NiO modified glassy carbon electrode.

CV data exposing nickel oxide modified surfaces to creatine showed no electrochemical catalytic activity, yet a reduction of capacitive current as concentration increased (Figure 7). This data, with the corrosion and SEM data, further supported the authors’ hypothesis that the guanidine moiety created a Schiff base that resulted in irreversible corrosion of the surface.

## 4. Conclusions

CuO, Fe_2_O_3_, and NiO surfaces were evaluated as catalysts for the electrochemical activation of various amino acids in a flow cell. While CuO and Fe_2_O_3_ did not yield strong current responses toward the amino acids, arginine had interesting effects on the NiO surface. The drastic changes in current shape and response of the NiO modified GCE in response to arginine was presumably due to accelerated degradation by complexation of the guanidine unit to Ni^2+^, which was supported by polarization resistance experiments.

Creatine, a structural analog of arginine, was evaluated with the NiO surface. Polarization resistance measurements demonstrated a similar rate of corrosion for creatine and arginine. That result supported the proposition that complexation of the catalyst to the guanidine moiety led to an increased solubility (delamination) of the NiO surface.

The authors encourage researchers interested in measuring arginine, and other guanidine moieties, to investigate more stable catalytic surfaces. To prevent the Schiff base formation, they propose two possible approaches. First, carbon nanotubes (e.g., multi-walled carbon nanotubes) and graphene, have the ability to host metal oxides with a Schiff base complex, which can impede the formation of additional Schiff base complexes from the solution [31]. Second, a negatively charged substrate, such as polyaniline and poly(*N*-isopropylacrylamide), can be used to host the nanoparticles and inhibit the Schiff base complex.

## Figures and Tables

**Table 1:** Effect of 1 mM arginine in 100 mM NaOH on corrosion of fresh NiO surfaces.

	Rate of Corrosion (10^−3^ Mils per Year)
1 mM Arginine in 100 mM NaOH	7.8 ± 0.6
400 μM Creatine in 100 mM NaOH	9.5 ± 1.2
100 mM NaOH (no analyte)	6.3 ± 0.7

## Supplementary Materials

The following are available online at http://www.mdpi.com/1424-8220/18/9/3144/s1, Figure S1: CVs of (A) alanine, (B) arginine, (C) serine, and (D) valine in 100 mM NaOH with a bare Pt working electrode. No electrochemical activity is observed. (B) For arginine, a degrease upon addition of amino acid is observed potentially due to a Schiff-base complex, Figure S2: Deposition of (A) Cu onto a Pt working electrode, (B) Fe onto GCE and (C) Ni onto GCE. The cathodic currents are indicative of reduction of the metal ion to the elemental metal. The decrease in current response with additional cycles is indicative of multiple layers of metal depositing on the surface, Figure S3: Passivation to (A) CuO onto a Pt working electrode, (B) Fe_2_O_3_ and (C) NiO onto GCE in 100 mM NaOH. A slight anodic current is occurred in the presence of 100 mM for the CuO surface and Fe_2_O_3_ surface, which decreased with iterative cycles due to the addition of more layers on the electrode surface, Figure S4: CVs of (A) alanine, (B) arginine, (C) serine, and (D) valine in PBS with an Fe_2_O_3_ modified GCE. Electrochemical activation of amino acids was not evident the CVs, presumably due to lack of chelation delamination of the surface from corrosion, Figure S5: CVs of (A) alanine, (B) arginine, (C) serine, and (D) valine in PBS with a NiO modified GCE. Electrochemical activation of amino acids was not evident the CVs, presumably due to lack of chelation delamination of the surface from corrosion, Figure S6: CVs of ferrocyanide in 100 mM NaOH from a (A) bare electrode and (B) after NiO modification. Three cycles of the same scan is displayed. No major changes of activity is observed, Figure S7: Polarization resistance plot of alanine on a NiO modified carbon QCM sensor. Corrosion Rate was found to be 1.960 × 10^−3^ mpy, Figure S8: Polarization resistance plot of arginine on a NiO modified carbon QCM sensor. Corrosion Rate was found to be 3.354 × 10^−3^ mpy; Figure S9: Polarization resistance plot of serine on a NiO modified carbon QCM sensor. Corrosion Rate was found to be 2.088 × 10^−3^ mpy, Figure S10: Polarization resistance of valine on a NiO modified carbon QCM sensor. Corrosion Rate was found to be 2.035 × 10^−3^ mpy, Table S1: Effect of 1 mM amino acid in 100 mM NaOH on corrosion of NiO surface after use in sensing experiments. Corrosion rate is highest with arginine at 3.4 × 10^−3^ mpy and lowest with a blank of 100 mM NaOH at 1.9 × 10^−3^ mpy. The rates reported in the manuscript are from a newly modified surface, which is different than the rates reported here, on an already used surface. The trend of the reported rates are still in agreement.
