# Supplementary material for: Evaluation of Metal Oxide Surface Catalysts for the Electrochemical Activation of Amino Acids
Source: Sensors (Basel). 2018 Sep 18;18(9):3144. doi: 10.3390/s18093144 (PMC6164431; doi:10.3390/s18093144)
Supplement: Supplementary file 1 [file sensors-18-03144-s001.pdf]

Supplemental Information for

Evaluation of Metal Oxide Surface Catalysts for the Electrochemical Activation of Amino Acids

Christian A. Tooley, Charles H. Gasperoni, Sabrina Marnoto and Jeffrey Mark Halpern *

Department of Chemical Engineering, University of New Hampshire, Durham, NH 03824, USA; christianatooley@gmail.com (C.A.T.), chg1001@wildcats.unh.edu (C.H.G.), smm1007@wildcats.unh.edu (S.M.)

* Correspondence: Jeffrey.Halpern@unh.edu(J.M.H.); Tel.: +01-603-862-5772

Supporting Information

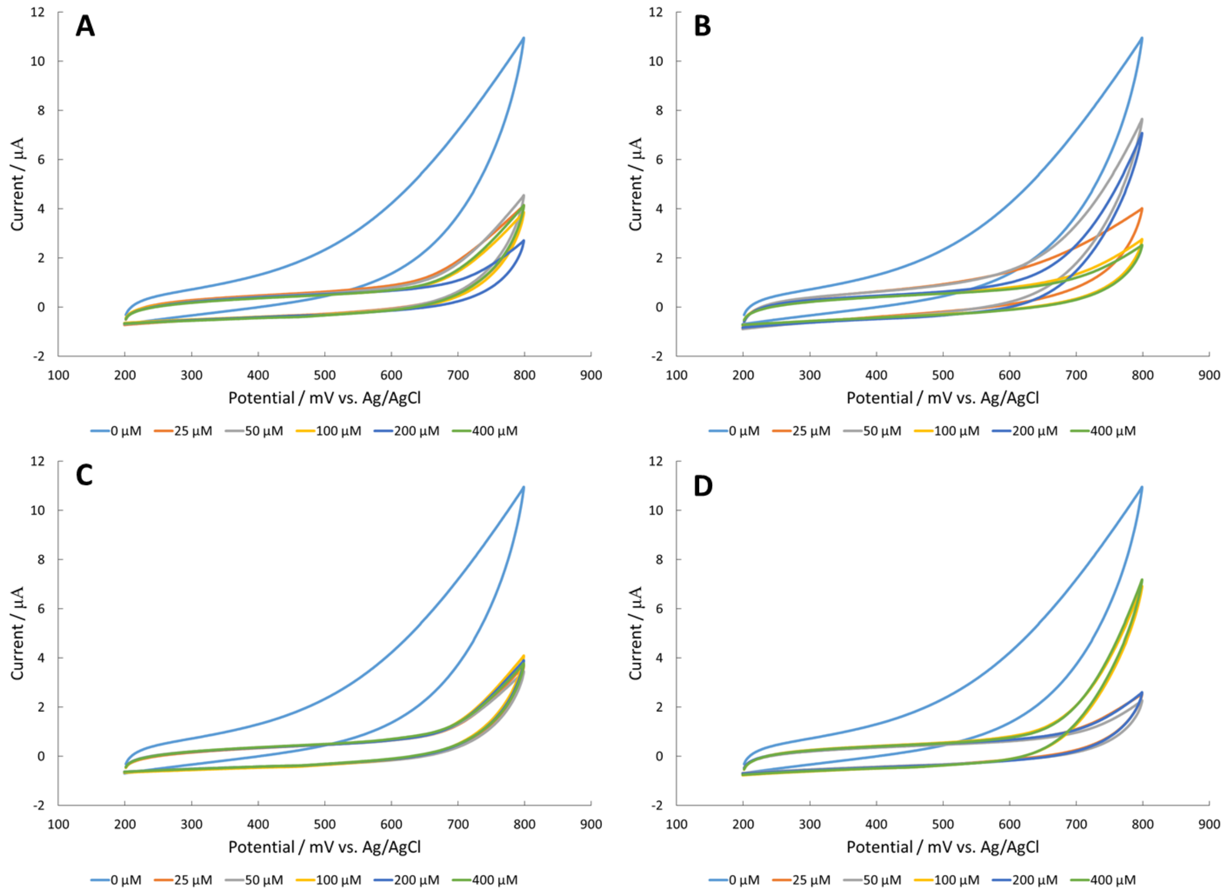

Figure S1. CVs of (**A**) alanine, (**B**) arginine, (**C**) serine, and (**D**) valine in 100 mM NaOH with a bare Pt working electrode. No electrochemical activity is observed. (**B**) For arginine, a degrease upon addition of amino acid is observed potentially due to a Schiff-base complex.

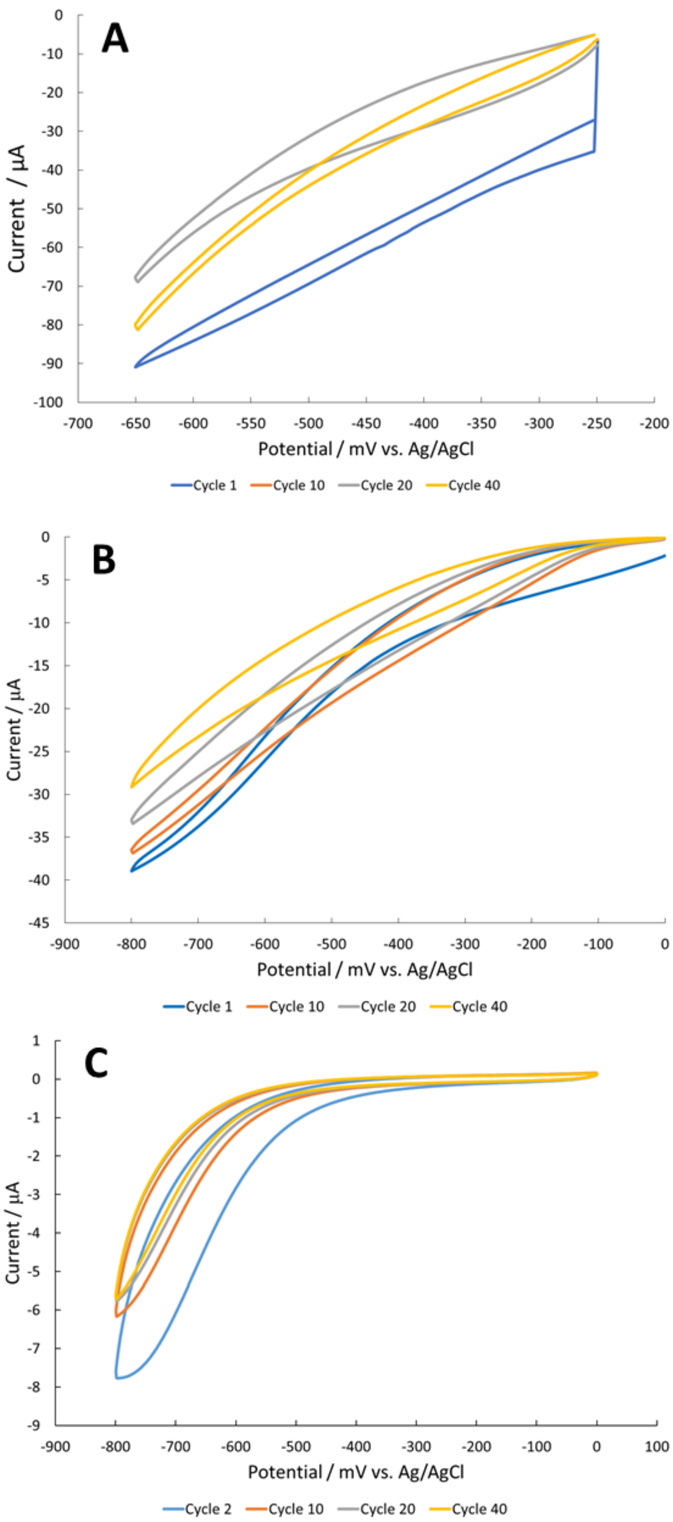

Figure S2. Deposition of (**A**) Cu onto a Pt working electrode, (**B**) Fe onto GCE and (**C**) Ni onto GCE. The cathodic currents are indicative of reduction of the metal ion to the elemental metal. The decrease in current response with additional cycles is indicative of multiple layers of metal depositing on the surface.

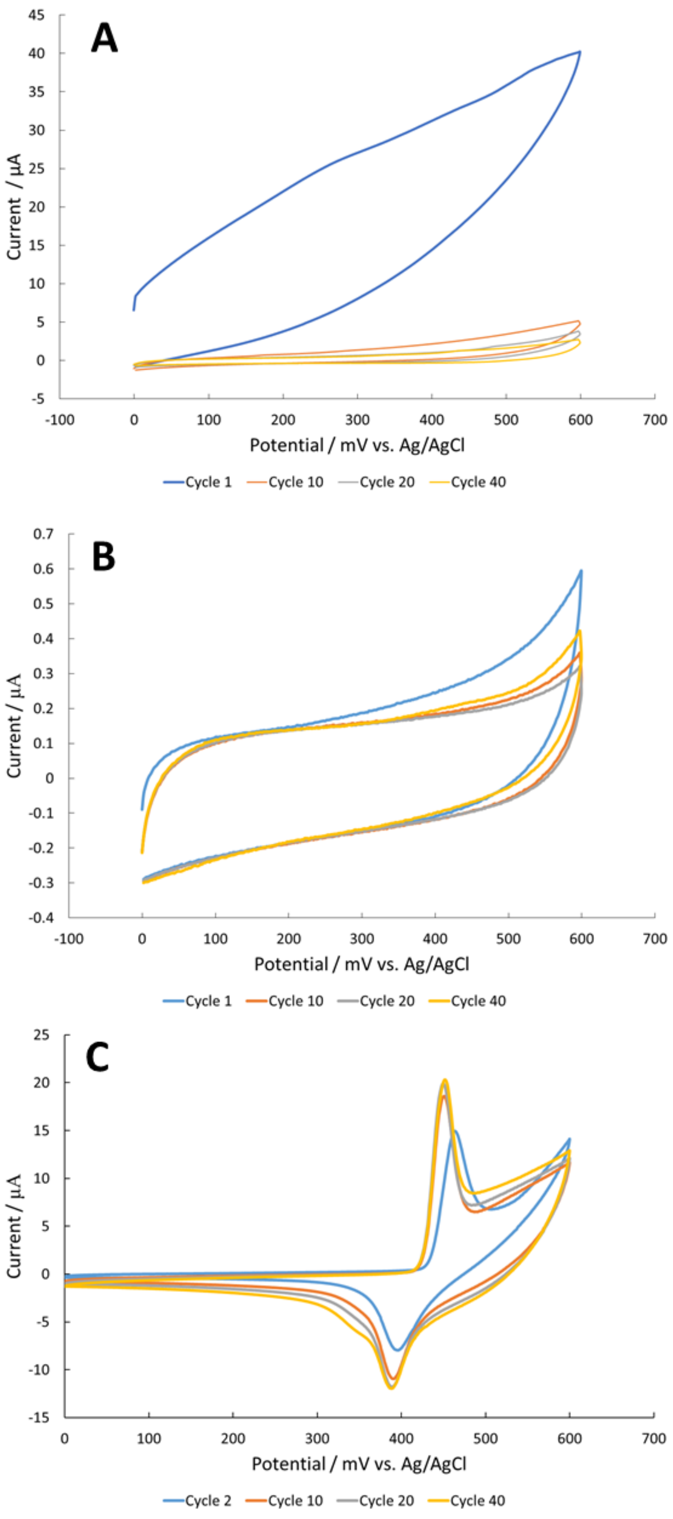

Figure S3. Passivation to (**A**) CuO onto a Pt working electrode, (**B**) Fe_2O_3 and (**C**) NiO onto GCE in 100 mM NaOH. A slight anodic current is occurred in the presence of 100 mM for the CuO surface and Fe_2O_3 surface, which decreased with iterative cycles due to the addition of more layers on the electrode surface.

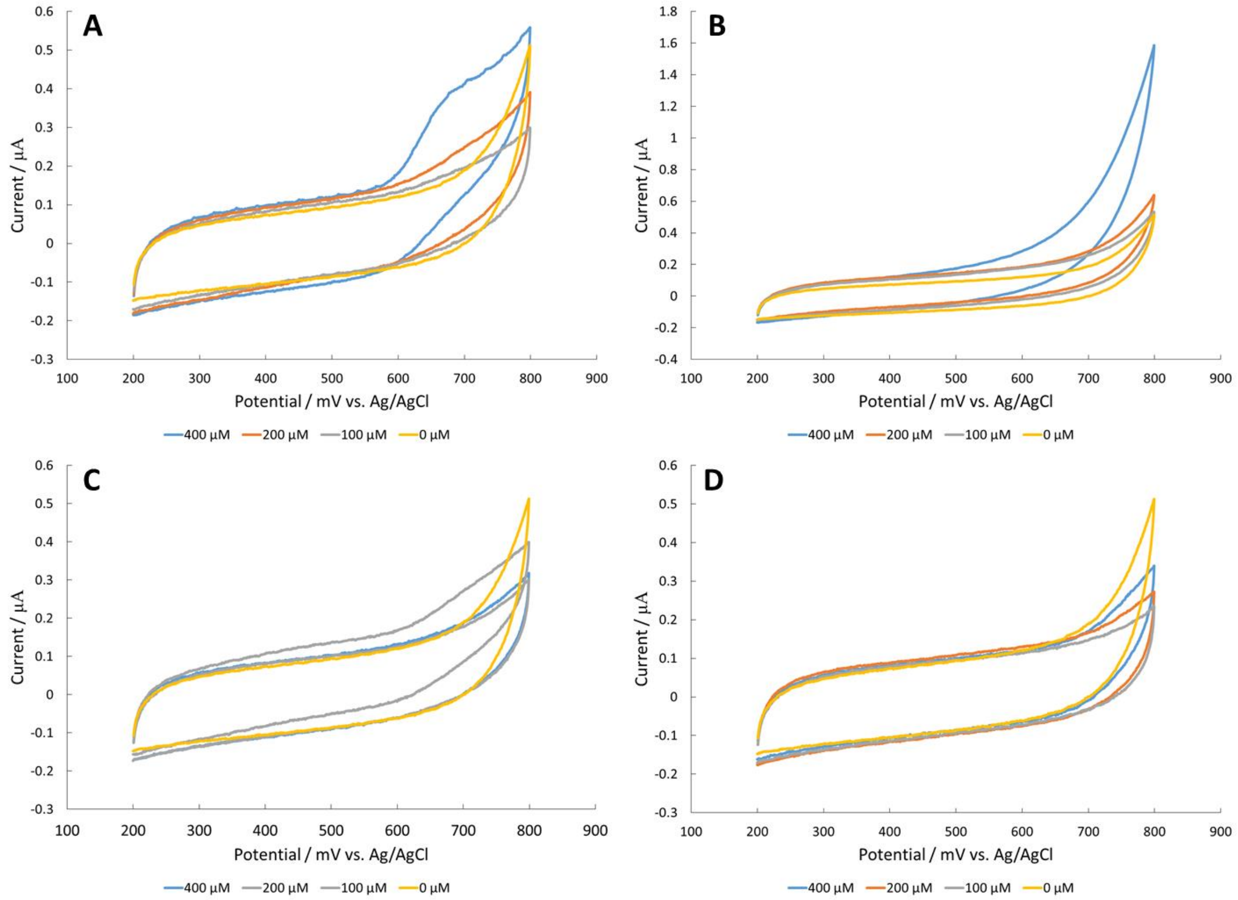

Figure S4. CVs of (**A**) alanine, (**B**) arginine, (**C**) serine, and (**D**) valine in PBS with an Fe_2O_3 modified GCE. Electrochemical activation of amino acids was not evident the CVs, presumably due to lack of chelation delamination of the surface from corrosion.

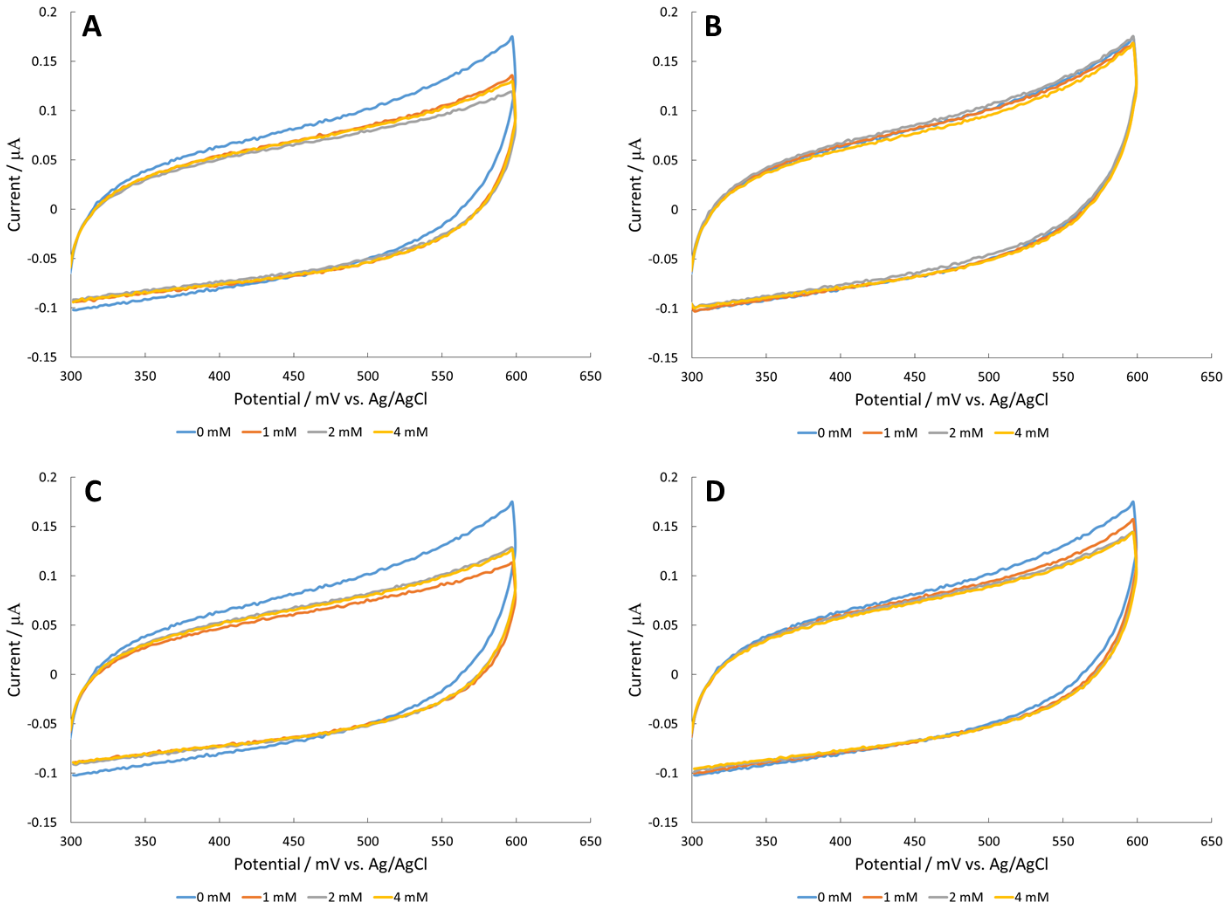

Figure S5. CVs of (**A**) alanine, (**B**) arginine, (**C**) serine, and (**D**) valine in PBS with a NiO modified GCE. Electrochemical activation of amino acids was not evident the CVs, presumably due to lack of chelation delamination of the surface from corrosion.

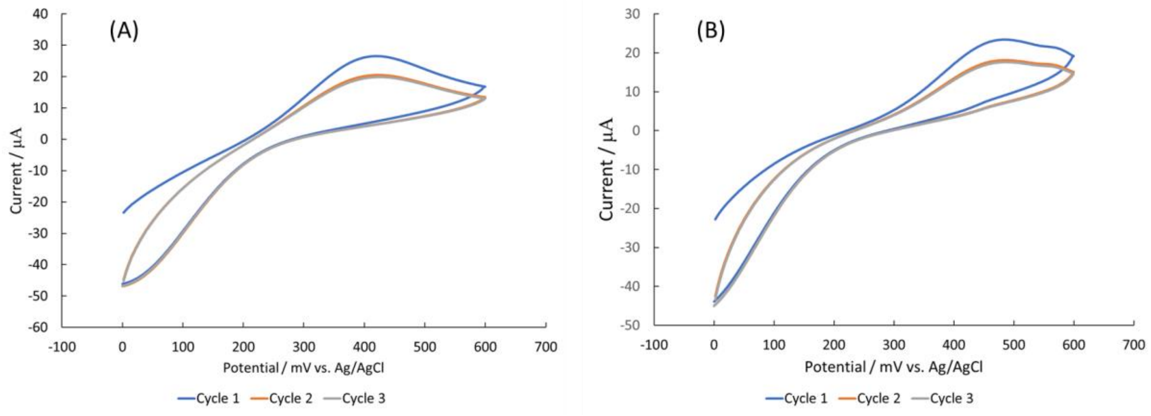

Figure S6. CVs of ferrocyanide in 100 mM NaOH from a (**A**) bare electrode and (**B**) after NiO modification. Three cycles of the same scan is displayed. No major changes of activity is observed.

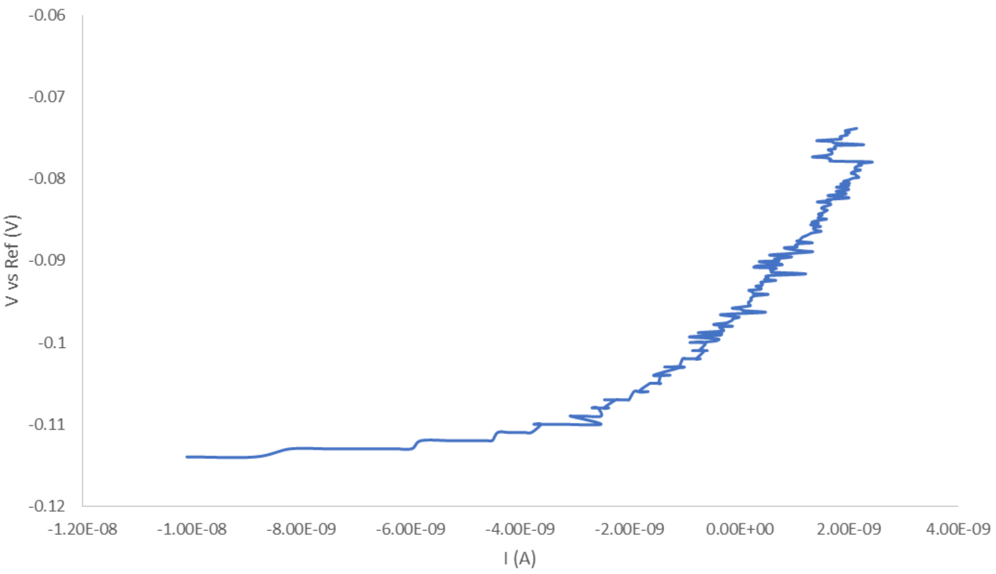

Figure S7. Polarization resistance plot of alanine on a NiO modified carbon QCM sensor. Corrosion Rate was found to be 1.960×10^{-3} mpy.

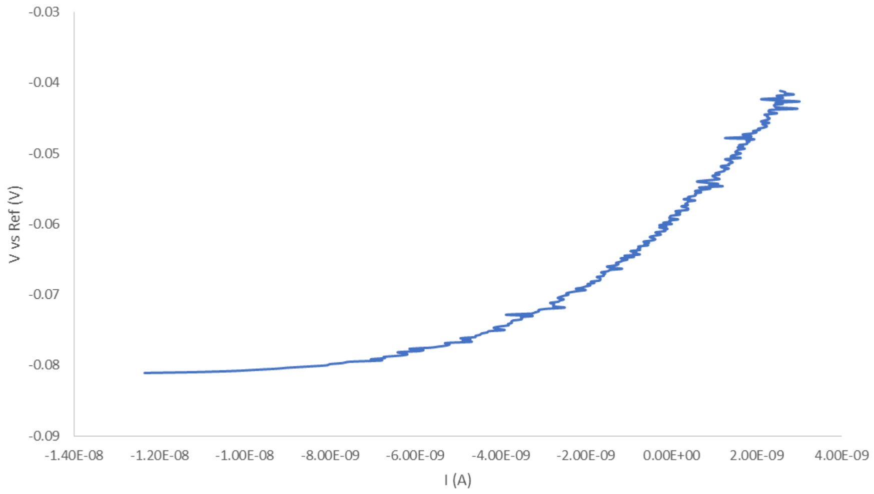

Figure S8. Polarization resistance plot of arginine on a NiO modified carbon QCM sensor. Corrosion Rate was found to be 3.354×10^{-3} mpy.

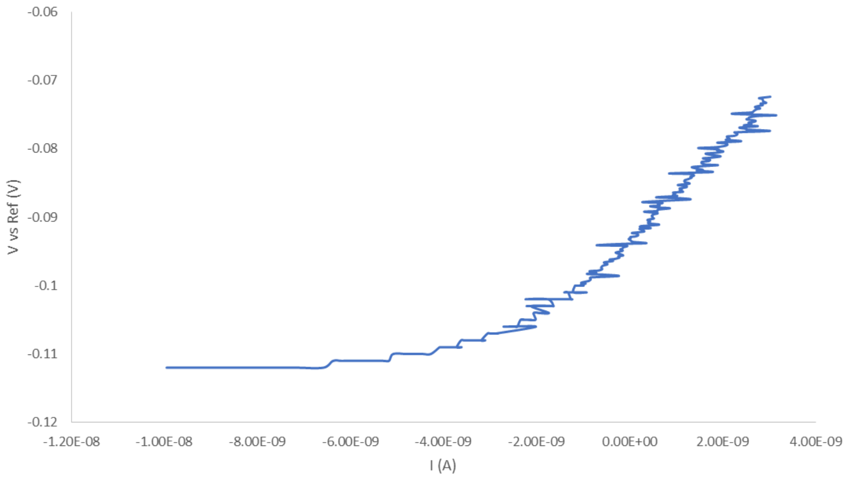

Figure S9. Polarization resistance plot of serine on a NiO modified carbon QCM sensor. Corrosion Rate was found to be 2.088 × 10^{-3} mpy.

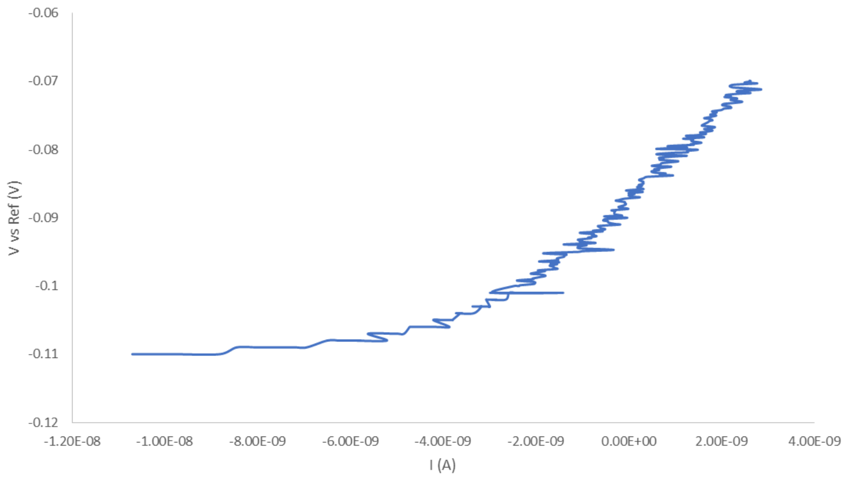

Figure S10. Polarization resistance of valine on a NiO modified carbon QCM sensor. Corrosion Rate was found to be 2.035 × 10^{-3} mpy.

Table S1. Effect of 1 mM amino acid in 100 mM NaOH on corrosion of NiO surface after use in sensing experiments. Corrosion rate is highest with arginine at 3.4 × 10^{-3} mpy and lowest with a blank of 100 mM NaOH at 1.9 × 10^{-3} mpy. The rates reported in the manuscript are from a newly modified surface, which is different than the rates reported here, on an already used surface. The trend of the reported rates are still in agreement.

Blank Sample (only 100 mM NaOH)	1.931 × 10^{-3} mpy
Arginine	3.354 × 10^{-3} mpy
Alanine	1.960 × 10^{-3} mpy
Valine	2.035 × 10^{-3} mpy
Serine	2.088 × 10^{-3} mpy

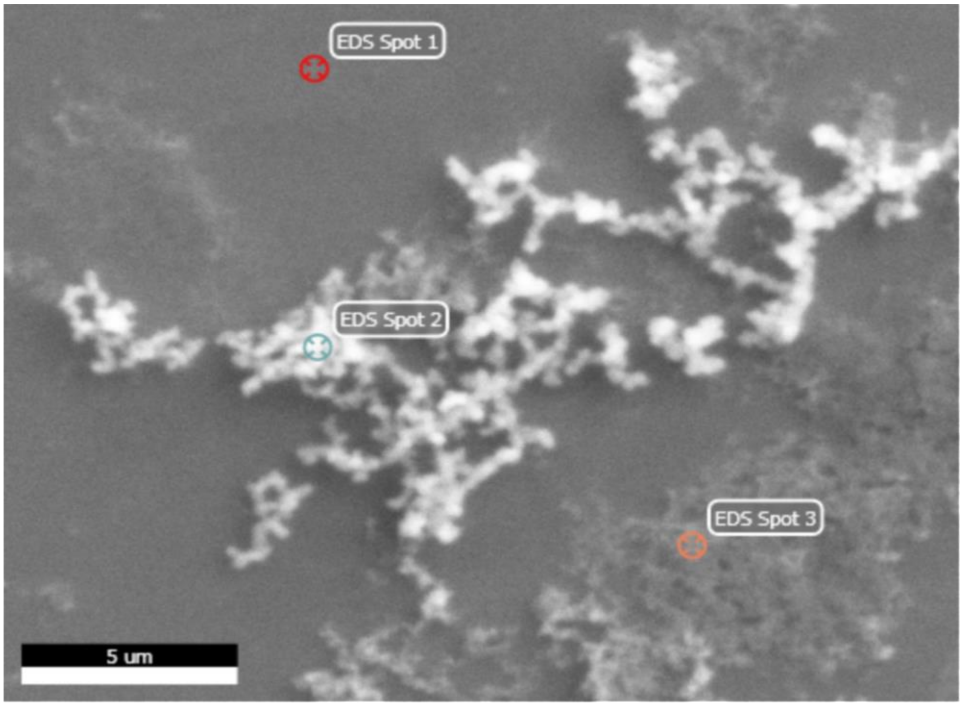

Spot 1				
Element	Weight %	Atomic %	Net Int.	Error %
C	15.43	58.50	441.36	6.18
O	0.61	1.72	25.09	17.40
Si	11.94	19.36	761.92	2.82
Au	66.54	15.38	1251.56	4.05
Ti	4.44	4.22	94.89	9.03
Ni	1.05	0.81	9.79	59.80

Spot 2				
Element	Weight %	Atomic %	Net Int.	Error %
C	12.37	33.91	458.61	5.59
O	18.00	37.05	1080.22	5.57
Na	2.81	4.02	149.18	8.05
Si	5.57	6.54	425.46	3.69
Au	41.38	6.92	977.82	4.95
Ti	3.38	2.32	90.30	8.62
Ni	16.48	9.24	181.27	6.03

Spot 3				
Element	Weight %	Atomic %	Net Int.	Error %
C	8.17	30.51	240.36	6.42
O	9.68	27.14	450.81	6.50
Na	2.41	4.70	112.60	6.97
Si	9.26	14.80	595.55	3.12
Au	59.25	13.50	1146.78	4.12
Ti	4.46	4.18	98.35	9.08
Ni	6.77	5.18	64.23	14.40

EDS report of nickel oxide nanoparticles on a carbon QCM chip. The QCM chip is layered, from bottom to top, with Si, Au, Ti, C. Nickel oxide is attached on-top of the carbon layer. Spot 1 is a control without any nickel content.

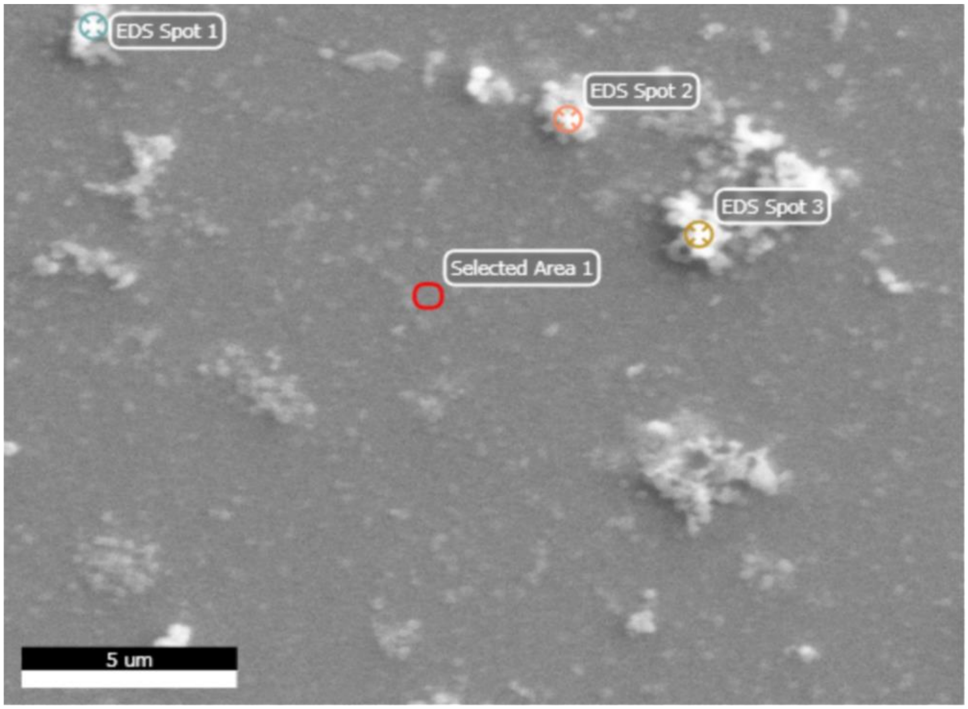

Selected Area				
Element	Weight %	Atomic %	Net Int.	Error %
C	16.33	57.61	547.73	6.26
O	0.40	1.05	19.88	22.18
Na	0.42	0.77	24.66	19.49
Si	14.92	22.52	1165.36	2.53
Au	62.67	13.49	1433.54	3.93
Ti	4.63	4.10	120.81	7.95
Ni	0.64	0.46	7.21	60.01

Spot 1				
Element	Weight %	Atomic %	Net Int.	Error %
C	15.43	44.20	626.98	5.73
O	11.16	23.99	693.37	6.38
Na	3.02	4.53	201.12	6.06
Si	9.43	11.56	844.26	2.97
Au	51.36	8.97	1383.16	4.41
Ti	3.94	2.83	119.29	9.39
Ni	3.91	2.29	49.68	14.97

Spot 2				
Element	Weight %	Atomic %	Net Int.	Error %
C	11.39	35.17	451.62	6.04
O	13.13	30.46	821.39	6.16
Na	2.60	4.20	169.78	6.43
Si	10.14	13.39	892.38	2.85
Au	54.00	10.17	1426.24	4.27
Ti	4.00	3.10	119.16	9.00
Ni	3.34	2.11	42.05	19.03

Spot 3				
Element	Weight %	Atomic %	Net Int.	Error %
C	13.41	38.10	545.89	5.82
O	14.02	29.90	885.74	6.12
Na	3.56	5.28	234.26	5.75
Si	8.27	10.05	736.98	2.98
Au	49.28	8.54	1330.00	4.36
Ti	3.75	2.67	113.67	9.01
Ni	4.72	2.74	59.69	17.02

EDS report of nickel oxide nanoparticles on a carbon QCM chip after corrosion experiments within PBS. The QCM chip is layered, from bottom to top, with Si, Au, Ti, C. Nickel oxide is attached on-top of the carbon layer. Selected area is a control without any nickel content.

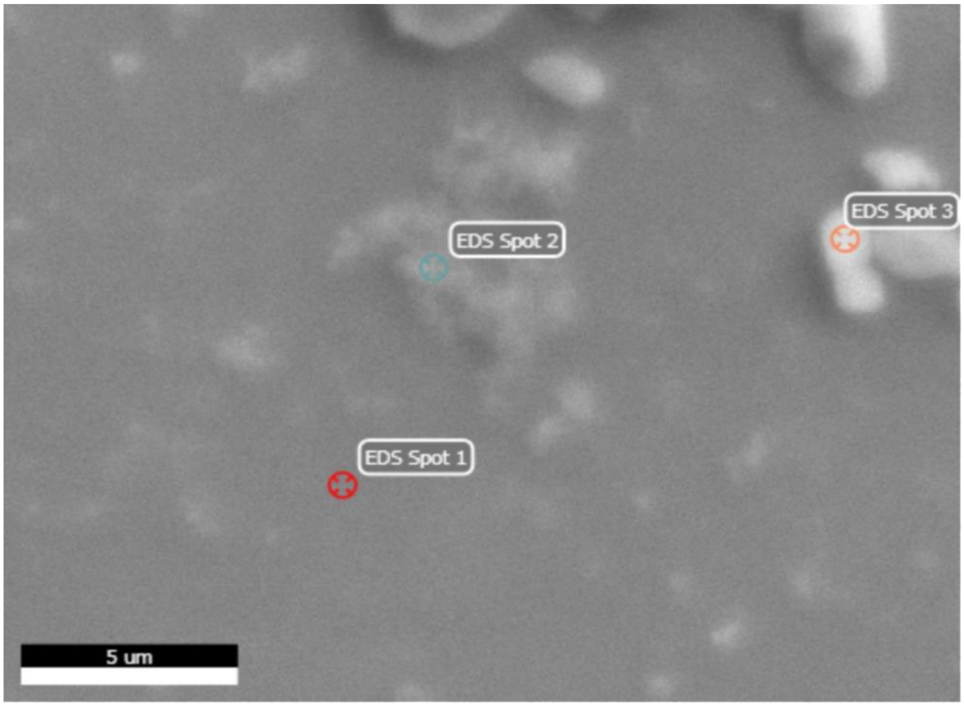

Spot 1				
Element	Weight %	Atomic %	Net Int.	Error %
C	15.50	59.04	557.03	6.07
O	0.13	0.36	6.56	75.83
Si	12.51	20.37	1010.75	2.64
Au	66.82	15.52	1588.52	3.92
Ti	4.40	4.20	119.00	8.68
Ni	0.65	0.51	7.68	61.99

Spot 2				
Element	Weight %	Atomic %	Net Int.	Error %
C	6.45	27.77	223.78	7.03
O	6.82	22.06	375.57	6.78
Na	1.40	1.23	46.04	8.70
Si	2.45	5.51	143.54	6.37
Au	11.48	21.14	899.87	2.90
Ti	67.07	17.61	1559.36	3.88
Ni	4.34	4.68	115.11	9.14

Spot 3				
Element	Weight %	Atomic %	Net Int.	Error %
C	3.92	13.88	101.19	10.41
O	0.32	0.85	20.00	20.67
NaK	19.45	35.98	1488.63	3.61
SiK	5.69	8.62	533.59	3.18
AuM	43.56	9.40	1275.08	4.43
ClK	23.53	28.22	1319.48	2.96
TiK	3.02	2.68	96.23	12.04
NiK	0.52	0.38	6.92	61.69

EDS report of nickel oxide nanoparticles on a carbon QCM chip after corrosion experiments with arginine. The QCM chip is layered, from bottom to top, with Si, Au, Ti, C. Nickel oxide is attached on-top of the carbon layer. Spot 1 is a control without any nickel content.
